# Laparoscopic Hartmann's reversal has better clinical outcomes compared to open surgery: An international multicenter cohort study involving 502 patients

**DOI:** 10.1002/hsr2.788

**Published:** 2022-09-01

**Authors:** Anwar Medellin Abueta, Nairo Javier Senejoa, Mauricio Pedraza Ciro, Lina Fory, Carlos Perez Rivera, Carlos Edmundo Martinez Jaramillo, Lina Maria Mateus Barbosa, Heinz Orlando Ibañez Varela, Javier A. Carrera, Rafael Garcia Duperly, Luis A Sanchez, Ivan David Lozada‐Martinez, Luis Felipe Cabrera‐Vargas, Andres Mendoza, Paulo Cabrera, Sebastian Sanchez Ussa, Cristian Paez, Steven D. Wexner, Victor Strassmann, Giovanna DaSilva, Salomone Di Saverio, Arianna Birindelli, Roberto Jose Rodríguez Florez, Abraham Kestenberg, Alexander Obando Rodallega, Juan Carlos Sánchez Robles, Carlos Adrian Niño Carrasco, Alessio Impagnatiello, Diletta Cassini, Gianandrea Baldazzi, Francesco Roscio, Gianluca Liotta, Pierluigi Marini, Daniel Gomez, Carlos Edgar Figueroa Avendaño, Daniela Moreno Villamizar, Laura Cabrera, Juan Carlos Reyes, Alexis Narvaez‐Rojas

**Affiliations:** ^1^ Department of Colorectal Surgery Fundación Santa Fe de Bogotá Bogotá Colombia; ^2^ Department of Colorectal Surgery Hospital Militar Central Bogotá Colombia; ^3^ Department of Surgery Universidad El Bosque Bogotá Colombia; ^4^ Department of General Surgery Hospital Militar Central Bogotá Colombia; ^5^ Department of General Surgery Fundación Cardioinfantil Bogotá Colombia; ^6^ Medical and Surgical Research Center Future Surgeons Chapter, Colombian Surgery Association Bogotá Colombia; ^7^ International Coalition on Surgical Research Universidad Nacional Autónoma de Nicaragua Managua Nicaragua; ^8^ Department of Surgery Fundación Santa Fe de Bogotá Bogotá Colombia; ^9^ Department of Surgery Pontificia Universidad Javeriana Bogotá Colombia; ^10^ Department of Surgery Fundación Universitaria Sanitas Bogotá Colombia; ^11^ Department of Colorectal Surgery Cleveland Clinic Florida Weston FL USA; ^12^ Emergency and General Surgery Department CA Pizzardi Maggiore Hospital Bologna Italy; ^13^ Department of Surgery Esine General Hospital ASST Valcamonica Italy; ^14^ Department of Colorectal Surgery Hospital Central Militar Ciudad de México México; ^15^ Department of Colorectal Surgery Fundación Clínica Valle del Lili Cali Colombia; ^16^ Department of Surgery San Caillo – Forlanini Hospital Rome Italy; ^17^ Complex Unit of General and Emergency Surgery Città di Sesto San Giovanni Hospital Milan Italy; ^18^ Department of General Surgery ASST Valle Olona Busto Arsizio Italy; ^19^ Department of General Surgery Hospital Universitario Mayor Méderi Bogotá Colombia

**Keywords:** colostomy, laparoscopy, laparotomy, operative surgical procedures, patient outcome assessment

## Abstract

**Background:**

Hartmann's procedure (HP) is used in surgical emergencies such as colonic perforation and colonic obstruction. “Temporary” colostomy performed during HP is not always reversed in part due to potential morbidity and mortality associated with reversal. There are several contributing factors for patients requiring a permanent colostomy following HP. Therefore, there is still some discussion about which technique to use. The aim of this study was to evaluate perioperative variables of patients undergoing Hartmann's reversal using a laparoscopic and open approach.

**Methods:**

The multicenter retrospective cohort study was done between January 2009 and December 2019 at 14 institutions globally. Patients who underwent Hartmann's reversal laparoscopic (LS) and open (OS) approaches were evaluated and compared. Sociodemographic, preoperative, intraoperative variables, and surgical outcomes were analyzed. The main outcomes evaluated were 30‐day mortality, length of stay, complications, and postoperative outcomes.

**Results:**

Five hundred and two patients (264 in the LS and 238 in the OS group) were included. The most prevalent sex was male in 53.7%, the most common indication was complicated diverticular disease in 69.9%, and 85% were American Society of Anesthesiologist (ASA) II‐III. Intraoperative complications were noted in 5.3% and 3.4% in the LS and OS groups, respectively. Small bowel injuries were the most common intraoperative injury in 8.3%, with a higher incidence in the OS group compared with the LS group (12.2% vs. 4.9%, *p* < 0.5). Inadvertent injuries were more common in the small bowel (3%) in the LS group. A total of 17.2% in the OS versus 13.3% in the LS group required intensive care unit (ICU) admission (*p* = 0.2). The most frequent postoperative complication was ileus (12.6% in OS vs. 9.8% in LS group, *p* = 0.4)). Reintervention was required mainly in the OS group (15.5% vs. 5.3% in LS group, *p* < 0.5); mortality rate was 1%.

**Conclusions:**

Laparoscopic Hartmann's reversal is safe and feasible, associated with superior clinical outcomes compared with open surgery.

## 1 INTRODUCTION

Hartmann's procedure (HP) may be performed for a myriad of colorectal diseases including large bowel perforation, obstruction, ischemic colitis, complicated diverticulitis, iatrogenic injuries, trauma, and cancer.^1^, ^2^, ^3^, ^4^, ^5^ Reversal of Hartmann's colostomy is a technically demanding procedure with 50% morbidity and 10% mortality rates.^6^ Moreover, 60% of patients will not undergo colostomy closure during the first postoperative year,^6^, ^7^ due to age, comorbidities, American Society of Anesthesiologists (ASA) score, or patient choice.^4,6^

The laparoscopic approach to Hartmann's colostomy reversal (LS) was first described about 30 years ago. Over the last two decades, studies have evaluated the minimally invasive approach to Hartmann's colostomy reversal. Data suggest lower rates of morbidity and mortality compared with open surgery (OS).^1^, ^2^ However, evidence shows an average high conversion rate of 25% due to multiple dense adhesions and difficulty in identifying the rectal stump.^7^, ^8^, ^9^ Recent evidence suggests that there are still gaps in the literature regarding analytical studies that have comparatively evaluated the outcomes of these two techniques.^10^, ^11^

It is necessary that the evidence be precise with respect to the usefulness and safety of each technique, since with the advance of technology and science, it is necessary to converge in the rational surgical practice, to save resources, reduce the risk of complications, guarantee functional capacity, and improve the quality of life.^12^, ^13^ Many studies report results from a single center. However, clinical and surgical outcomes need to be evaluated globally.^10^, ^11^ Therefore, the aim of this study was to compare OS and LS approaches for Hartmann colostomy reversal with emphasis on assessing clinical and surgical outcomes.

## 2 MATERIALS AND METHODS

### 2.1 Study design

The study has been reported in line with the strengthening the reporting of cohort studies in surgery answer criteria.^14^ An international multicenter retrospective cohort study of 14 institutions around the world was carried out. All patients who underwent Hartmann's colostomy reversal between January 2009 and December 2019 were identified. All patients underwent either open or laparoscopic surgery depending on the surgeon's experience, preference, and resources.

### 2.2 Patient selection and data collection

Patients over 16 years of age who underwent a Hartmann's colostomy reversal procedure using either OS or LS were included. Early postoperative follow‐up within the first 30 days was undertaken by either outpatient clinic appointments or telephone interviews. Data from the participating surgeons at each of the 14 centers were collected and entered into a single database, maintained by the lead investigator. A table of standard definitions and operationalizations was created and shared with each participating institution.

Age, sex, body mass index (BMI), comorbidities, preoperative history of radiotherapy, and/or chemotherapy, American Society of Anesthesiologist (ASA) score, operative time, blood loss, time interval since HP, intraoperative findings, postoperative complications, ileus, intensive care unit (ICU) admission, length of hospital stay (LOS), reintervention, mortality, and other short‐term results during the first 30 days after surgery were recorded.

All the patients underwent bowel preparation (including >1 enema to empty the rectal stump) approximately 24 h before surgery; preoperative broad‐spectrum parenteral antibiotics were administered.

### 2.3 Primary endpoints

The following primary endpoints were evaluated to determine any impact of the method of surgical intervention (OS vs. LS):

1. Postoperative complications and outcomes, including return to surgery and time to first bowel movement.
2. LOS, defined as the number of days from postoperative until discharge.
3. 30‐day postoperative mortality.

Primary endpoints were independently evaluated as binary outcomes. All associations of the surgical approach with an outcome were examined in univariable (unadjusted) and multivariable (adjusted) logistic regression analyses.

### 2.4 Surgical technique

**Figure 1:**
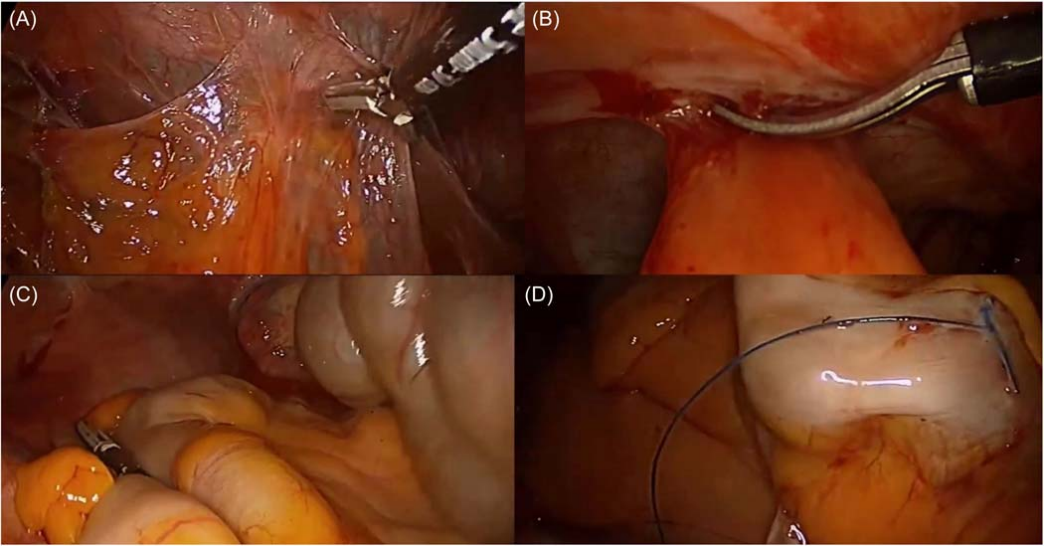
(A) The first port is located in the transition of the right upper and inferior quadrants with the mid clavicular line using the open Hasson technique to avoid incidental bowel injuries. (B) Laparoscopic adhesiolysis is performed using an advanced bipolar sealing energy device and laparoscopic scissors using the shaving technique to avoid accidental and missed bowel injuries. (C) Laparoscopic identification and dissection of the rectal stump. (D) Hybrid open and laparoscopic end colostomy resection.

All the surgeons based their surgical technique for laparoscopic colostomy reversal after left colectomy with end colostomy (Hartmann procedure) in the procedure described by Brac et al.^15^ (Figure 1), limiting the variation of the surgical technique among the participants surgeons, in order not to affect the homogeneity of the results obtained. The step‐by‐step surgical procedure is summarized as follows: 1. Patient is located in French position; 2. The ports are located in the right side of the abdomen; 3. The first port is located in the transition of the right upper and inferior quadrants with the mid clavicular line using the open Hasson technique to avoid incidental bowel injuries; 4. Under direct vision, using a 30° laparoscope, the surgeons located two work ports, one of 5 mm in the upper right quadrant and one of 12 mm in the inferior right quadrant; 5. Laparoscopic adhesiolysis is performed using an advanced bipolar sealing energy device and laparoscopic scissors using the shaving technique to avoid accidental and missed bowel injuries; 6. Laparoscopic mobilization of the left colon splenic flexure and the transverse colon; 7. Laparoscopic identification and dissection of the rectal stump; 8. Hybrid open and laparoscopic end colostomy resection; 9. Performance of laparoscopic colorectal anastomosis using a circular transrectal stapler; and finally, 10. Pneumatic test for colorectal anastomosis.

### 2.5 Statistical analysis

Clinical findings or characteristics based on the surgical approach were assessed using a Student's *t* test and Mann–Whitney *U* test to compare the means between groups for normally distributed and nonnormally distributed data, respectively. The *χ* ^2^ test was used to compare proportions/frequencies between groups. Primary endpoints were evaluated independently as binary outcomes. In the univariate analysis, a *χ* ^2^ test was used. Variables with a *p* ≤ 0.1 in the appropriate univariable model were selected for inclusion in the corresponding multivariable logistic or linear regression models. In the multivariate analysis, a stepwise logistic regression was used. Statistical significance was considered as *p* ≤ 0.05. Statistical analysis was undertaken using Microsoft Excel 2003 (Microsoft Corporation) and R 4.0.1 binary for macOS 10.13 (High Sierra).

### 2.6 Ethical statements

The study was approved by each of the institutions' ethics review boards. The protocol was implemented in accordance with the Declaration of Helsinki^16^ and Good Clinical Practice guidelines.^17^ The ethics committee exempted the collection of informed consent due to the retrospective nature of the study and the minimal risk.

## 3 RESULTS

Five hundred and two patients were included (264 in LS group vs. 238 in OS group). Patients were predominantly male (270/502 [53.7%], LS: 140/264 [53%] vs. OS: 130/238 [54.6%]), the most frequent indication was inflammatory pathology (351/502 [69.9%], LS: 197/264 [74.6%] vs. OS: 154/238 [64.7%]), followed by cancer (114/502 [22.7%]) and trauma (37/502 [7.3%]; *p* < 0.05). The most common prior surgery was appendicectomy (58 [11.5%], LS: 43 [16.2%] versus OS: 15 [6.3%]; *p* < 0.05) (Table 1).

**Table 1:** Sociodemographic and clinical characteristics of patients who underwent laparoscopic versus open surgery Hartmann's reversal

Variable	All (*n* = 502)	LS Group (*n* = 264)	OS Group (*n* = 238)	*p value*
Median age, years (IQR)		50 (18)	53 (20)	<0.05
Sex				
Female	232 (46.2)	124 (46.9)	108 (45.3)	0.7891
Male	270 (53.7)	140 (53.0)	130 (54.6)	
Median BMI, Kg/m^2^ (%)				
<18.5	21 (4.1)	12 (4.5)	9 (3.7)	0.07586
18.5–24.9	201 (40.0)	92 (34.8)	109 (45.7)	
25–29.9	194 (38.6)	114 (43.1)	80 (33.6)	
30–34.9	67 (13.3)	39 (14.7)	28 (11.7)	
35–39.9	15 (2.9)	5 (1.8)	10 (4.2)	
>40	4 (0.8)	2 (0.7)	2 (0.8)	
Hartmann's indication, *n* (%)				
Cancer	114 (22.7)	54 (20.4)	60 (25.2)	<0.05
No cancer	351 (69.9)	197 (74.6)	154 (64.7)	
Trauma	37 (7.3)	13 (4.9)	24 (10.0)	
Prior abdominal surgeries, *n* (%)				
Appendectomy	58 (11.5)	43 (16.2)	15 (6.3)	<0.05
Cholecystectomy	37 (7.3)	21 (7.9)	16 (6.7)	0.7215
Peritoneal lavage	29 (5.7)	15 (5.6)	14 (5.8)	1
Bowel obstruction due to cancer	21 (4.1)	9 (3.4)	12 (5.0)	0.4907
Hernia repair	5 (0.9)	1 (0.3)	4 (1.6)	0.3093
Other	77 (15.3)	27 (10.2)	50 (21.0)	<0.05
ASA Classification, *n* (%)				
I	66 (13.1)	36 (13.6)	30 (12.6)	0.499
II	291 (57.9)	147 (55.6)	144 (60.5)	
III	137 (27.2)	78 (29.5)	59 (24.7)	
IV	8 (1.5)	3 (1.1)	5 (2.1)	
Prior abdominal surgery, *n* (%)				
1	296 (58.9)	169 (64.0)	127 (53.3)	<0.05
2	97 (19.3)	52 (19.6)	45 (18.9)	
3	53 (10.5)	29 (10.9)	24 (10.0)	
4	33 (6.5)	9 (3.4)	24 (10.0)	
Preoperative ventral hernia, *n* (%)	143 (28.4)	65 (24.6)	78 (32.7)	0.05466
Patient comorbidities, *n* (%)				
Hypertension	183 (36.4)	97 (36.7)	86 (36.1)	0.9613
Coronary artery disease	35 (6.9)	15 (5.6)	20 (8.4)	0.3077
Pulmonary disease	32 (6.3)	19 (7.1)	13 (5.4)	0.5409
Diabetes type 2	60 (11.9)	30 (11.3)	30 (12.6)	0.7715
Immunosuppression	20 (3.98)	7 (2.6)	13 (5.4)	0.1678
Congestive heart failure	41 (8.16)	18 (6.8)	23 (9.6)	0.3177
Anticoagulation	27 (5.37)	18 (6.8)	9 (3.7)	0.1909
Others	153 (30.47)	72 (27.2)	81 (34.0)	0.1221
>2 comorbidities	130 (25.89)	63 (23.8)	67 (28.1)	0.3207
None	94 (18.72)	35 (13.2)	59 (24.7)	<0.05
Neoadjuvant or adjuvant treatment, *n* (%)				
Neoadjuvant radiotherapy	3 (0.6)	1 (0.3)	2 (0.8)	0.1184
Adjuvant radiotherapy	2 (0.4)	0 (0)	2 (0.8)	
Neoadjuvant chemotherapy	13 (2.5)	3 (1.1)	10 (4.2)	
Adjuvant chemotherapy	57 (11.3)	32 (12.1)	25 (10.5)	
Neoadjuvant radio‐chemotherapy	7 (1.3)	3 (1.1)	4 (1.6)	
Adjuvant radio‐chemotherapy	1 (0.2)	1 (0.4)	0 (0)	
None	413 (82.2)	221 (83.7)	192 (80.6)	

Abbreviations: ASA, American Society of Anesthesiologists; BMI, body mass index; IQR, interquartile range; LS, laparoscopic; OS, open surgery.

A total of 291 (57.9%) patients were ASA II, and 137 (27.9%) were ASA III. The most frequent comorbidities were arterial hypertension [183/502 (36.4%)] and type 2 diabetes mellitus (60/502 [11.9%]); 130/502 (25.8%) patients had more than two comorbidities.

### 3.1 Intraoperative variables

No statistically significant difference was found in the type of anastomosis between the two groups. The most frequent iatrogenic injury reported was a small bowel enterotomy. Divided in the ones with intraoperative diagnosis, representing 42 patients (8.3%) (LS: 13 [4.9%] versus OS: 29 [12.1%]; *p* < 0.05), and those ones with postoperative diagnosis, present in 11 patients (4.3%) (LS: 8 [3.0%] versus OS: 3 [1.3%], *p* = 0.06).

Fifty‐seven (21.5%) patients in the OS group had their procedure converted due to technical difficulty in 38 (14.3%), fecal contamination in 3 (1.1%), active bleeding in 1 (0.3%), and other reasons in 15 (5.6%) (*p* < 0.05). Overall, intraoperative bleeding volume was <100 cc in 338 (67%) patients (LS: 189/264 [71.6%] vs. OS: 149/238 [62%]; *p* < 0.05). The most frequent intraoperative complication was injury to adjacent structures in 15/502 (3.0%) patients (LS: 10/264 [3.8%] vs. OS: 5/238 [2.1%]; *p* = 0.522) (Table 2).

**Table 2:** Distribution of intraoperative surgical variables

Variable	All (*n* = 502)	LS group (*n* = 264)	OS group (*n* = 238)	*p value*
Type of anastomosis, *n* (%)				
Stapled end‐to‐end	373 (74.3)	205 (77.7)	168 (70.6)	<0.05
Stapled side‐to‐end	83 (16.5)	57 (21.6)	26 (10.9)	
Hand‐sewn end‐to‐end	4 (0.8)	0 (0)	4 (1.6)	
Hand‐sewn end‐to‐side	1 (0.2)	0 (0)	1 (0.4)	
Stapled side‐to‐side	40 (7.9)	2 (0.76)	38 (16)	
Hand‐sewn side‐to‐side	1 (0.2)	0 (0)	1 (0.4)	
Intraoperative injury, *n* (%)				
Small bowel	42 (8.3)	13 (4.9)	29 (12.2)	<0.05
Colon	3 (0.6)	1 (0.4)	2 (0.8)	
Urinary tract	5 (1)	4 (1.5)	1 (0.4)	
Reproductive organs	0 (0)	0 (0)	0 (0)	
Other	10 (1.9)	1 (0.4)	9 (3.7)	
No	451 (89.8)	245 (92.8)	206 (86.6)	
Iatrogenic missed injury, *n* (%)				
Small bowel	11 (2.1)	8 (3)	3 (1.2)	0.06796
Colon	2 (0.4)	0 (0)	2 (0.8)	
Urinary tract	1 (0.2)	0 (0)	1 (0.4)	
Reproductive organs	3 (0.6)	3 (1.1)	0 (0)	
Other	9 (1.7)	7 (2.6)	2 (0.8)	
No	476 (94.8)	246 (93.2)	230 (96.6)	
Median surgical time (IQR)		187 (70)	180 (113)	0.45326
Median blood loss, ml (%)				
0–100 ml	338 (67.3)	189 (71.6)	149 (62.6)	<0.05
101–200 ml	87 (17.3)	46 (17.4)	41 (17.2)	
201–500 ml	58 (11.6)	22 (8.3)	36 (15.1)	
>500 ml	19 (3.78)	7 (2.6)	12 (5)	
Intraoperative complications, *n* (%)				
Bleeding	7 (1.3)	4 (1.5)	3 (1.2)	0.5221
Injury to neighboring structures	15 (2.9)	10 (3.7)	5 (2.1)	
None	480 (95.6)	250 (94.7)	230 (96.6)	

Abbreviation: IQR, interquartile range.

### 3.2 Postoperative outcomes

A total of 76 patients (15.1%) required postoperative ICU admission (LS 35 [13.2%] vs. OS 41 [17.2%]; *p* = 0.26]). The time to resumption of an oral diet was higher in the OS group with a median of 2.2 days versus 1.7 days in the LS group (*p* < 0.05).

The most frequent postoperative complication was ileus, 56 (11.15%) (LS 26 [9.8%] vs. OS 30 [12.6%]; *p* = 0.4), followed by superficial surgical site infection (SSI) in 36 (7.2%) (LS: 19 [7.1%] vs. OS 17 [7.1%]; *p* = 1.0). LOS was shorter in the LS group at a median of 5.2 days versus 6.1 days in the OS group. Patients with LS required fewer reoperations (14/264 [5.3%]) versus those in the OS group (37/238 [15.5%]; *p* < 0.05) (Table 3).

**Table 3:** Distribution of postoperative outcomes in the study population

Variable	All (*n* = 502)	LS group (*n* = 264)	OS group (*n* = 238)	*p* value
ICU required, *n* (%)				
No	426 (84.9)	229 (86.7)	197 (82.8)	0.2652
Yes	76 (15.1)	35 (13.3)	41 (17.2)	
Mean time to postoperative oral tolerance, days (IQR)		1.73 (2)	2.20 (3)	<0.05
Mean time to first passage of stool, days (IQR)		2.54 (1)	2.94 (2)	<0.05
Postoperative morbidity, *n* (%)				
Ileus	56 (11.2)	26 (9.8)	30 (12.6)	0.4022
Pneumonia	8 (1.5)	3 (1.1)	5 (2.1)	0.6137
Unplanned mechanical ventilation	11 (2.1)	3 (1.1)	8 (3.3)	0.163
Cardiovascular	5 (1)	2 (0.7)	3 (1.2)	0.9072
Acute renal failure	4 (0.8)	1 (0.3)	3 (1.2)	0.544
Thromboembolic event	2 (0.4)	1 (0.3)	1 (0.4)	1
Superficial SSI	36 (7.1)	19 (7.2)	17 (7.1)	1
Deep SSI	8 (1.5)	2 (0.7)	6 (2.5)	0.2207
Sepsis	11 (2.1)	6 (2.2)	5 (2.1)	1
Ventral hernia	29 (5.7)	18 (6.8)	11 (4.6)	0.3889
Evisceration	3 (0.6)	1 (0.3)	2 (0.8)	0.9282
Intestinal fistula	7 (1.3)	2 (0.7)	5 (2.1)	0.3679
Anastomotic bleeding	6 (1.2)	2 (0.7)	4 (1.6)	0.5898
Other	12 (2.3)	9 (3.4)	3 (1.2)	0.2002
None	334 (66.5)	182 (68.9)	152 (63.9)	0.2678
Mean length of hospital stay, days (IQR)		5.2 (3)	6.1 (4)	1
Need for reintervention, *n* (%)	51 (10.2)	14 (5.3)	37 (15.5)	<0.05
30‐day mortality, *n* (%)	5 (1)	2 (0.7)	3 (1.2)	0.9072

Abbreviations: ICU, intensive care unit; IQR, interquartile range; SSI, surgical site infection.

### 3.3 Mortality in OS versus LS

Protective factors for mortality in OS were side‐to‐side handsewn anastomosis (odds ratio [OR]: 0.83; 95% confidence interval [CI]: 0.73–0.95, *p* < 0.05) and intraoperative injuries (OR: 0.71; 95% CI: 0.66–0.77, *p* < 0.05). Risk factors were iatrogenic detected colon injuries (OR: 1.3; 95% CI: 1.14–1.4; *p* < 0.05), iatrogenic missed colon injuries (OR: 1.13; 95% CI: 1.0–1.3, *p* < 0.05), and postoperative intestinal fistula (OR: 1.15; 95% CI: 1.1–1.2, *p* < 0.05) (Table 4).

**Table 4:** Associations between perioperative and postoperative variables with mortality, operative complications and length of stay in the open surgery group

Variable	OR	95% CI	*p value*
Mortality			
Days to postoperative stool evacuation	0.99	0.98–0.99	<0.05
Colonic injury	1.26	1.14–1.39	<0.05
Iatrogenic missed colonic injury	1.13	1.00–1.28	<0.05
Injuries^a^	0.71	0.66–0.77	<0.05
Postoperative intestinal fistula	1.15	1.09–1.22	<0.05
Operative complications			
Iatrogenic missed injuries (a)	0.60	0.43–0.82	<0.05
Bleeding 200–500 cc	0.89	0.84–0.95	<0.05
Postoperative pneumonia	1.16	1.01–1.35	<0.05
Length of stay (days)			
Days to postoperative stool evacuation	2.12	1.2–3.5	<0.05
Superficial SSI	1.66	1.08–2.74	<0.05
Deep SSI	14.4	3.29–17.7	<0.05
Reintervention	9.71	7.18–13.1	<0.05

Abbreviation: SSI, surgical site infection.

^a^ Small bowel and genitourinary.

Protective factors for mortality in LS were no iatrogenic missed colon injury (OR: 0.91; 95% CI: 0.86–0.96, *p* < 0.05). Risk factors were length of ileus (OR: 1.0; 95% CI: 1.004–1.019, *p* < 0.05), LOS (OR: 1.003; 95% CI: 1.001–1.004, *p* < 0.05), time to surgery longer than 24 months (OR: 1.12; 95% CI: 1.06–1.17, *p* < 0.05), end‐to‐side handsewn anastomosis (OR: 1.03; 95% CI: 1.00–1.05, *p* < 0.05), postoperative pneumonia (OR: 1.31; 95% CI: 1.16–1.480, *p* < 0.05); intestinal fistula (OR: 1.24; 95% CI: 1.11–1.39, *p* < 0.05), and reintervention (OR: 1.06; 95% CI: 1.01–1.11, *p* < 0.05) (Table 5).

**Table 5:** Associations between perioperative and postoperative variables with mortality, operative complications, conversion, and length of stay in the laparoscopic group

Variable	OR	95% CI	*p* value
Mortality			
Days to postoperative oral tolerance	1.01	1.00–1.01	<0.05
Length of stay	1.00	1.00–1.00	<0.05
Waiting surgical time > 24 months	1.12	1.06–1.17	<0.05
Injuries (a)	0.91	0.86–0.96	<0.05
Postoperative pneumonia	1.31	1.16–1.48	<0.05
Postoperative fistula	1.24	1.11–1.39	<0.05
Reintervention	1.06	1.01–1.11	<0.05
Conversion			
Surgical time	0.99	0.99–0.99	<0.05
Waiting surgical time > 24 month	3.2	1.49–6.88	<0.05
Operative complications			
Surgical time	1.00	1.00–1.00	<0.05
Surgical waiting surgical time 7 to 12 months	0.93	0.88–0.98	<0.05
Urinary tract injury	1.25	1.00–1.55	<0.05
Unplanned mechanical ventilation	1.36	1.02–1.81	<0.05
Length of hospital stay			
Days to postoperative oral tolerance	1.89	1.11–3.24	<0.05
Inadvertent injury to reproductive organs	3.54	1.2–14	<0.05

^a^ Colonic, small bowel, and genitourinary.

### 3.4 Operative complications in OS vs. LS

In the OS group, protective factors were end‐to‐end handsewn anastomosis (OR: 0.79; 95% CI: 0.68–0.92, *p* < 0.05). Risk factors were postoperative pneumonia (OR: 1.16; 95% CI: 1.01–1.35, *p* < 0.05) and a thromboembolic event (OR: 2.29; 95% CI: 1.69–3.10, *p* < 0.05).

In the LS group, protective factors for morbidity were 7 to 12 months since first intervention (OR: 0.93; 95% CI: 0.88–0.98, *p* < 0.05). Risk factors were surgical time (OR: 1.00; 95% CI: 1.00007–1.0008, *p* < 0.05), iatrogenic injury of the urinary tract (OR: 1.25; 95% CI: 1.00–1.55, *p* < 0.05), and unplanned mechanical ventilation (OR: 1.36; 95% CI: 1.02–1.81, *p* < 0.05).

### 3.5 Length of stay in OS versus LS

In the OS group, risk factors were days to resumption of normal bowel function (OR: 2.12; 95% CI: 1.2–3.5, *p* < 0.05), superficial SSI (OR: 1.66; 95% CI: 1.08–2.74, *p* < 0.05), deep SSI (OR: 14.4; 95% CI: 3.29–17.7, *p* < 0.05), and reintervention (OR: 9.71; 95% CI: 7.18–13.1, *p* < 0.05). In the LS group, risk factors were tolerance to oral intake (OR: 2.12; 95% CI: 1.2–3.5, *p* < 0.05), superficial SSI (OR: 1.66; 95% CI: 1.08–2.74, *p* < 0.05), deep SSI (OR: 14.4; 95% CI: 3.29–17.7, *p* < 0.05), and reintervention (OR: 9.71; 95% CI: 7.18–13.1, *p* < 0.05).

### 3.6 Conversion to OS

The only protective factor against conversion to OS was surgical time (OR: 0.99; 95% CI: 0.995–0.999, *p* < 0.05). The only risk factor was time to surgery > 24 months (OR: 3.2; 95% CI: 1.49–6.88, *p* < 0.05).

## 4 DISCUSSION

In 1923, Henri Hartmann first described HP as a rectal cancer and this technique is still used in emergency colorectal surgery for patients with significant comorbidity and a higher risk of anastomotic dehiscence. One of the problems with this technique is that these patients require a two‐stage surgical procedure to restore normal intestinal transit. The second stage has shown significant morbidity rates, giving reason to doubt the possibility of a colostomy reversal.^1^, ^2^, ^9^ In addition, the use of the laparoscopic approach for this entity has been limited due to technical difficulties associated with severe postoperative adhesions.^1^, ^2^, ^9^

Although initially HP was considered a temporary measure, Hartmann's reversal rate is relatively low with nearly 40% of patients not being reversed.^7^, ^8^ Currently, the most common causes of emergency HP are acute perforated diverticulitis and sigmoid volvulus. Recent studies report the implementation of primary colorectal anastomosis, possibly with proximal diverting ileostomy, in the acute care surgery setting. However, HP still remains a valid option for unstable or critically ill patients who are at high risk for anastomotic leak. Hartmann's reversal procedure has traditionally been performed with an OS approach.^9^, ^18^

The myriad of benefits of minimally invasive surgery (MIS) including less pain, lower SSI, and early postoperative recovery, have made it increasingly used in all surgical fields, and Hartmann's colostomy reversal procedure is no exception. Our study is the first retrospective international multicenter study to compare the results of LS versus OS Hartmann's reversal procedure. Laparoscopic Hartmann's colostomy reversal is a totally feasible and safe procedure and is associated with low complication rates and mortality.^9^, ^18^

Fifty‐two percent of patients in our study underwent laparoscopic Hartmann's reversal, showing an increased use of the laparoscopic approach in this procedure compared with previous reports. According to a study from the American College of Surgeons' National Surgical Quality Improvement Program (ACS‐NSQIP) database in 2015, only 17.6% of Hartmann's reversal procedures were performed using a laparoscopic approach.^4^

Nevertheless, the majority of the reports include only small numbers of patients. To our knowledge, our comparative retrospective worldwide multicenter study represents the largest experience in laparoscopic Hartmann's reversal. Van de Wall et al.^2^ reported lower complications rates as well as shorter hospital stays. However, in our study, the LS group had a mean hospital length of stay of 5.2 days versus 6.1 days for the OS group, without statistical significance. Similarly, our results showed no statistically significant differences between the LS and OS groups in terms of age, indication of HP, prior abdominal surgery, time of surgery, and absence of comorbidities. Park et al.^19^ reported similar results and concluded that laparoscopic Hartmann's reversal is a safe and feasible procedure associated with superior clinical outcomes.

Inadvertent injuries of adjacent organs were more frequent in the LS group versus the OS group in our study, although this finding did not reach statistical significance. This finding is in keeping with the published literature and confirms that inadvertent injuries to adjacent structures are more common in laparoscopic versus open colorectal procedures.^19^, ^20^

Different types of anatomoses were performed in both groups in our study. However, this heterogeneity did not show any relationship with complications or anastomotic leak rates. Our conversion to OS rate in the LS was 21.5%, which is lower than reported in the literature.^20^, ^21^, ^22^ The only risk factor for conversion was time for surgery longer than 24 months. We could not identify technical difficulties such as multiple adhesions, number of previous procedures, or severity of disease that necessitated the HP as risk factors.

The reported literature shows lower postoperative morbidity rates in the laparoscopic approach for Hartmann's colostomy reversal compared with OS (between 30% and 50% for OS and 15% for the laparoscopic approach).^20^, ^21^, ^22^ Our study found a 30‐day morbidity of 31.1% for LS versus 36.1% for OS, without statistical significance, which is different than in the reported literature.^23^, ^24^, ^25^ The most frequent postoperative complication was ileus for both groups at 11.1% (*n* = 56) [LS 26 (9.84%) vs. OS 30 (12.6%); *p* = 0.4]. These results differ from the series of Haughn et al.^26^ in which the most frequent morbidity was colostomy wound infection.

Melkonian et al.^25^ reported a single‐center experience with no mortality in either MIS Hartmann's reversal or OS group. However, the mortality rate following open Hartmann's reversal in recent series varies between 0.6% and 1.7%.^24^, ^26^ Our study shows a lower 30‐day mortality rate for the LS group of 0.7% versus 1.2% for the OS group, without statistical significance.

In our study, the surgical time was higher in the LS than in the OS group at 187 versus 180 min, respectively. This added time was due to the need for complete laparoscopic adhesiolysis, although this additional time did not seem to adversely impact postoperative outcomes and was in fact a protective factor for conversion to OS. This finding is similar to the results reported by Celentano et al.^27^ in a systematic review and meta‐analysis that included 13 studies comparing 862 patients (403 LS vs. 459 OS) with no significant difference in operating time, in contrast with previous series that reported a shorter operative time in the LS group.^27^

Resumption of oral intake was faster in the LS versus the OS group in our study. Ileus was the most frequent postoperative complication and was more frequent in the OS group. It has been widely demonstrated that OS is associated with longer postoperative ileus and increased costs, due to a longer length of hospital stay.^21^, ^22^ Another factor for a rise in treatment costs is perioperative infection.^25^, ^26^ However, we were unable to identify any significant differences in SSI between the OS and LS groups in our study. We believe this finding was due to the fact that the most frequent site of SSI in both groups were at the stoma site.

The main limitations of this study are that it is a retrospective, nonrandomized observational study without a standardized surgery protocol. These limitations may have led to selection bias. In an attempt to address this potential problem, we included a clear definition of the inclusion criteria and the resulting outcomes to ensure minimal bias inherent to this type of study. In addition, the number of cases per center was heterogeneous and may affect interpretation of the results. Nonetheless, to our knowledge, this study is the first cohort evaluation of these patients from different countries. This methodology potentially allows for extrapolation of the results to a worldwide population with a sufficient and significant sample size to determine the best surgical approach for Hartmann's reversal procedure.

### 4.1 Conclusions

Laparoscopic Hartmann's reversal is a safe and feasible procedure associated with superior clinical outcomes compared with an OS approach. This minimally invasive approach has low morbidity and faster recovery. Based on these results, laparoscopy should be considered as the approach of choice for Hartmann's colostomy reversal procedure if appropriately skilled staff and surgeons are available.

## AUTHOR CONTRIBUTIONS

All authors contributed to the conceptualization; data curation; formal analysis; investigation; methodology; validation; writing—original draft; writing—review & editing.

## CONFLICT OF INTEREST

The authors declare no conflict of interest.

## TRANSPARENCY STATEMENT

The lead author affirms that this manuscript is an honest, accurate, and transparent account of the study being reported; that no important aspects of the study have been omitted; and that any discrepancies from the study as planned (and, if relevant, registered) have been explained.

## ETHICS STATEMENT

This study was approved by each of the institutions’ ethics review boards.

## Data Availability

The datasets used and/or analyzed during the current study are available from the corresponding author upon reasonable request. All authors have read and approved the final version of the manuscript. The corresponding author had full access to all of the data in this study and takes complete responsibility for the integrity of the data and the accuracy of the data analysis.
